# Comparing Two Treatment Approaches for Patients with Type 1 Diabetes During Aerobic Exercise: a Randomised, Crossover Study

**DOI:** 10.1186/s40798-021-00319-5

**Published:** 2021-04-29

**Authors:** Varun Vartak, Lynne Chepulis, Matthew Driller, Ryan G. Paul

**Affiliations:** 1 Te Huataki Waiora, School of Health, University of Waikato, Hamilton, New Zealand; 2 Waikato Medical Research Centre, Te Huataki Waiora, School of Health, University of Waikato, Hamilton, New Zealand; 3 Sport and Exercise, School of Allied Health, Human Services & Sport, La Trobe University, Melbourne, Australia; 4 Waikato Regional Diabetes Service, Waikato District Health Board, Hamilton, New Zealand

**Keywords:** Hypoglycaemia, Hyperglycaemia, Glycaemic control, Insulin, Carbohydrates

## Abstract

**Background:**

In a randomised, counterbalanced, crossover design, eight men with type 1 diabetes (T1D; mean ± SD age, 27.6 ± 11.4 years) reduced insulin (INS) by 50% of their normal dose or consumed carbohydrates equivalent to 1 g of carbohydrate per kilogramme of their body weight without the usual insulin bolus (CARBS) over two sessions, held a week apart. Each session included standardised meals, a 45-min treadmill walk at 7.24 km h^−1^ and a 6-min walk test (6MWT). Rate of perceived exertion (RPE), blood glucose, ketone and lactate measures were taken before, during and immediately after the aerobic exercise. The distance covered in metres and the predicted VO_2_ max (mL kg^−1^ min^−1^) were also calculated for the 6MWT.

**Results:**

Participants completing the INS intervention spent more time in normoglycaemia (242 ± 135 min vs 88 ± 132 min; *P* < 0.01) and less time in hyperglycaemia (41 ± 95 min vs 154 ± 125 min; *P* = 0.01) as compared to the CARBS intervention. Mild hypoglycaemia occurred in two participants during INS and no participants during CARBS. Furthermore, there was no significant difference for blood lactate, ketone, RPE, distance covered and predicted VO_2_ max between interventions.

**Conclusion:**

Based on this pilot study, INS intervention appears to be the best approach for maintaining blood glucose levels in those with T1D during aerobic exercise, though this does need evaluation in other groups, including women, children and those with suboptimal glycaemic control.

**Trial Registration:**

Australian New Zealand Clinical Trial Registry, ACTRN12619001397101p. Registered 09 September 2019.

## Key Points

- Reducing insulin appears to be more effective than eating carbohydrates without the usual insulin bolus for maintaining glycaemic control during aerobic exercise.
- Reduced insulin was the preferred intervention for 75% of participants when evaluated using a questionnaire with a closed-ended question (which intervention, if either, would you prefer for future exercise?).

## Introduction

Exercise is a cornerstone of management of type 1 diabetes (T1D) as it can aid with glycaemic control, reduce cardiovascular disease and improve quality of life [1–3]. However, many patients with T1D are concerned about undertaking exercise because of the risk of developing hypoglycaemia and how to adjust their insulin or carbohydrate intake to prevent aberrations in their glycaemic control [3, 4]. Indeed, in a systematic review of 10 studies, the effects of physical exercise and recovery in T1D were shown to be dependent on exercise scheduling, duration and intensity of the exercise, prior carbohydrate consumption, insulin therapy, pre-exercise glucose levels and cardiovascular fitness [5]. However, with an adequate knowledge of metabolic responses and appropriate diabetes self-management, patients with T1D should be able to overcome their fear of exercise-induced dysglycaemia [3, 4].

Guidelines recommend that to prevent exercise-induced hypoglycaemia, patients with T1D must either reduce their basal insulin doses or consume carbohydrates without co-administration of insulin prior to commencing exercise [6, 7]. However, whilst research does indicate that both strategies can independently support glycaemic control during exercise [8], knowledge of whether one strategy is better than the other is lacking and only limited peer-reviewed studies have compared both approaches during exercise within the same experimental design [9–11]. Thus, the aim of the current study was to evaluate the effects of these two approaches on glycaemic control (time in normo-, hypo- and hyperglycaemia) and performance in a single bout of aerobic exercise. We also measured the effects of the two interventions on predicted VO_2_ max in a 6-min maximum walk test (6MWT), and on patient-perceived performance.

## Methods

### Participants

Men with T1D were recruited directly from the Waikato Regional Diabetes Service (WRDS, Hamilton, New Zealand) between September and November 2019. To be eligible, all participants needed to have had T1D for at least 2 years, be aged 18–60 years, have a recent HbA1c (within the last 3 months) of 45–90 mmol/mol (6.3–13.9%), be able to walk/run unaided for 45 min at a speed of 7.24 km h^−1^, and be fully competent with diabetes self-management. Participants were excluded if they were taking testosterone therapy, had any comorbidities that would impact on their ability to complete the study (e.g. cardiovascular disease, cerebrovascular disease, severe diabetic retinopathy, nephropathy, neuropathy), were taking medications that altered heart rate (e.g. beta-blockers) or were currently using closed-loop insulin pump therapy. All potential participants were screened at the WRDS Clinic and upon recruitment were required to attend the clinic again 38–48 hours prior to the first test session to have a Flash Glucose monitoring (FGM) device (Abbott Freestyle Libre, Chicago, IL, USA) inserted to allow for continuous glucose monitoring [12]. All participants were able to wear the Freestyle Libre device for 14 days from the date of insertion. The study was successfully registered under the Australian New Zealand Clinical Trial Registry (ACTRN12619001397101p) and was approved by the New Zealand Health and Disability Ethics Committee (ref 19/NTB/175).

### Study Design

This study was a randomised, counterbalanced, crossover design, in which men with T1D evaluated two different interventions for managing glycaemia whilst undertaking a pre-defined exercise protocol. The two sessions were carried out a week apart at the University of Waikato gymnasium in a controlled, indoor environment with meals (breakfast and lunch) provided. The sessions were conducted by a research team consisting of a diabetologist/endocrinologist, a clinical research specialist, a clinical nurse, a sport physiologist and a health research student. The night before each session, participants were required to consume a meal that contained at least 1 g of carbohydrate/kg of body weight [6], and they were asked to eat the same meal before both intervention sessions. Consumption of alcohol, caffeine-based beverages and performing strenuous exercise were prohibited during the 12 h before each aerobic exercise session. Participants then fasted following dinner on the previous day until 9 am the following morning.

The two interventions assessed were (i) reducing their insulin (INS) or (ii) consuming carbohydrates without the usual insulin bolus (CARBS) along with the breakfast meal. The same breakfast meal was provided at 9 am in both groups and consisted of bananas, apple juice and 100 % isolate protein powder (Musclepharm COMBAT, New Zealand), with quantities individualised to contain 0.66 g of carbohydrate/kg of body weight and 3.375 kcal/kg of body weight, meeting both the requirements of exercise carbohydrates guidelines [6] and calculated energy expenditure using the Browning Walking Metabolic Prediction equation [13]. Dietitian advice was sought to ensure all meals, including the meal the night before the exercise, met the required recommendations. The accurate intake of breakfast was observed by the investigators and all participants confirmed that they had consumed the recommended meal the night prior.

Participants were either on multiple daily injections of insulin (MDI) or continuous subcutaneous infusion of insulin (CSII; insulin pump therapy). All patients on MDI were on a long-acting basal insulin (insulin glargine) and rapid-acting bolus insulin (either insulin aspart or insulin lispro). All patients on insulin pump therapy used a continuous infusion of insulin aspart. During the INS intervention, participants on MDI did not alter their basal insulin but halved their bolus insulin of their normal dose immediately before consuming the breakfast meal as a single bolus as per the consensus guidelines [7]. Those participants in the INS intervention on CSII started a 50% temporary basal rate reduction 90 min before aerobic exercise and halved their normal bolus dose for their meal. The 50% temporary basal rate reduction stopped 60 min after completion of the aerobic exercise [7]. The participants in the CARBS intervention consumed their breakfast meal without the administration of any bolus insulin and did not alter their basal insulin [6]. Throughout the test sessions, the participants were not allowed to further adjust their insulin doses or carbohydrates intake. Any adjustments (required or accidental) would trigger the cessation of the session for this participant.

All participants began the aerobic exercise session at 10:00 am and were required to walk on a motorised treadmill (Life Fitness, Chicago, IL; USA) at a fixed speed of 7.24 km h^−1^ (4.5 miles/h) at a 1% gradient for 45 min. According to the ACSM classification, this would be classified as “vigorous” intensity exercise [14]. Ten minutes following completion of the treadmill walk, participants then performed the 6MWT [15] by walking as quickly as possible for 6 min around the perimeter of an inside arena. This test is considered a validated measure of predicted VO_2_ max, and this was calculated using the equation as follows [16]. VO_2_ max (mL kg^−1^ min^−1^) = 70.161 + (0.023 × 6-min walk distance covered [m]) − (0.276 × weight [kg]) − (6.79 × sex, where male = 0, female = 1) − (0.193 × resting heart rate [BPM]) − (0.191 × age [years]). All participants were allowed to be comfortably seated on their arrival at the lab for 5 min. Their resting heart rate was measured using the heart rate monitor (RS800cx, Polar Electro Oy, Finland). For the 6MWT, distances were marked off every 3 m, and the total distance walked in the 6 min was recorded. Approximately 60 min after completion of the exercise activities, participants had lunch (One Square Meal, OSM: Queenstown, New Zealand), individualised to consist of 1 g of carbohydrate/kg of body weight and administered their normal dose of bolus insulin [7]. Water was allowed ad libitum throughout each session and no additional adjustments (including self-adjustment) of insulin or carbohydrate were allowed until 3 h after lunch.

Capillary blood glucose was initially measured before breakfast. Capillary glucose and lactate were measured immediately at the start of the 45-min aerobic exercise, every 15 min during the aerobic exercise, at the end of the 45-min aerobic exercise, within 1–3 min after completion of the 6MWT [17] and before lunch. Blood ketone level measurements were taken at the beginning and the end of the 45-min aerobic exercise and within 1–3 min after completion of the 6MWT. Blood lactate was measured using a lactate analyser (Lactate Pro 2, Arkray, Japan). Glucose levels were monitored continuously using the FGM device at every 15 min for the entire duration the session (time range from 0900 to 1500), and also via capillary sampling (Abbott Optium glucose strips) to ensure there was no significant lag in changes between interstitial and blood glucose levels during physical activity [12]. Self-perceived rate of exertion (RPE) was also assessed every 15 min during the aerobic exercise and immediately following the 6MWT using the Borg’s RPE scale [18]. After completion of both sessions, participants were asked which intervention, if either, they preferred for future exercise.

### Statistical Analysis

Descriptive statistics and data are expressed as mean ± standard deviation (SD). Paired *t*-tests (two-tailed) were performed on blood glucose levels, blood ketone, blood lactate, RPE, distance covered and the predicted VO_2_ max for the two interventions. Repeated measures ANOVA was performed to compare time in the normal blood glucose range (primary outcome), time in hyperglycaemia and time in hypoglycaemia for INS and CARBS. Thresholds for normal range or normoglycaemia were 3.9–10 mmol/L, mild hyperglycaemia between 10.1 and 13.9 mmol/L, severe hyperglycaemia ≥ 14 mmol/L, mild hypoglycaemia < 3.9 mmol/L and significant hypoglycaemia (< 3 mmol/L) [19]. The time in each range was calculated from the FGM but importantly there were no significant differences or lag between capillary and interstitial glucose levels in any participants. Statistical significance was accepted at a level of *P* < 0.05.

## Results

Eight men with T1D participated in the study (age, 27.6 ± 11.4 years; weight, 91.8 ± 11.0 kg; BMI, 26.6 ± 2.2 kg m^2^; HbA1c, 55.1 ± 7.4 mmol/mol) and all were able to complete both sessions without the need for additional insulin correction. Six participants were on multiple daily injections of insulin (MDI) and two participants on CSII.

**Fig. 1:**
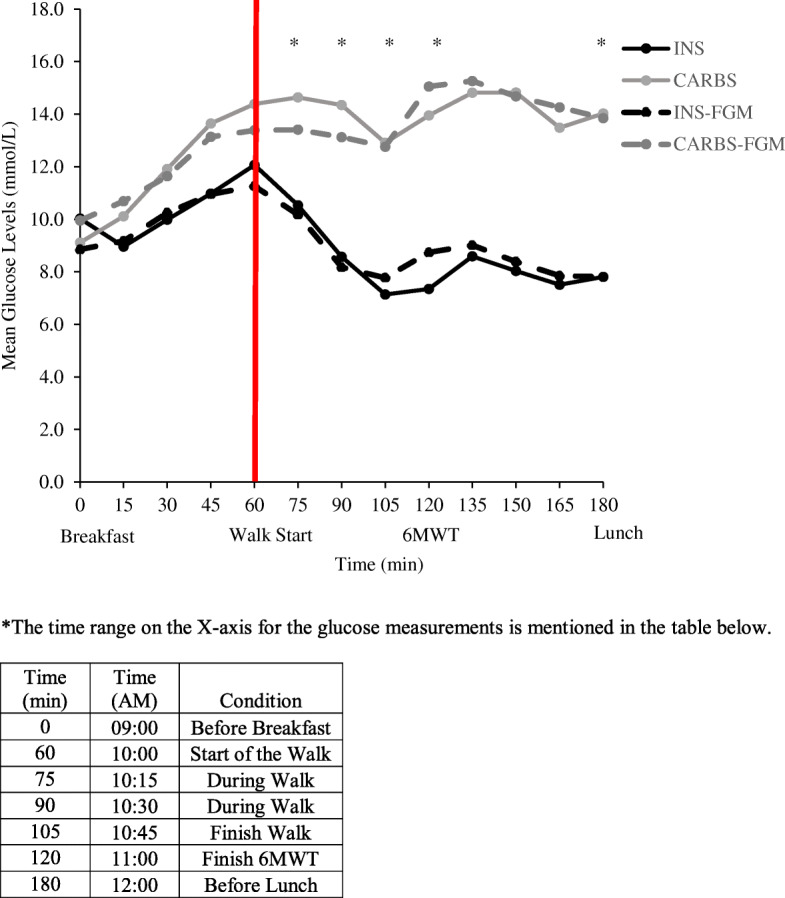
The effects of reducing bolus insulin with (INS) vs consuming carbs without insulin (CARBS) on the capillary blood glucose levels and on the interstitial blood glucose levels (FGM). *P < 0.05 between INS and CARBS

Overall, participants completing the INS intervention spent more time in normoglycaemia (242 ± 135 min vs 88 ± 132 min; *P* < 0.01) and less time in severe hyperglycaemia (41 ± 95 min vs 154 ± 125 min; *P* = 0.01) compared to the CARBS intervention (Fig. 1). The time in mild hyperglycaemia was also halved during the INS intervention (mean 66 ± 76 min vs 133 ± 111 min), though this was not statistically different (*P* = 0.23). Two participants on CSII experienced mild hypoglycaemia during INS and participants on MDI experienced no hypoglycaemia during INS. Also, no hypoglycaemia was observed in any participants during CARBS (26 ± 45 min vs 0 min; *P* = 0.17). Mean blood glucose levels remained significantly higher during CARBS than INS from 15 min into the aerobic exercise until the duration of the study, despite a varied yet comparable blood glucose level just prior to breakfast (Fig. 1). From the repeated measures ANOVA, there was a statistically significant interaction between the two interventions (INS and CARBS) and time spent in each blood glucose level range *F* (3, 18) = 6.574, *P* < 0.001.

There were no significant differences in blood lactate, ketone levels or rate of perceived exertion (RPE) during the treadmill test at any timepoints between interventions (all *P* > 0.05). However, there was a trend for participants completing the INS intervention to walk further in the 6MWT than the CARBS intervention (mean ± SD 777.9 ± 319.9 m vs 724.6 ± 302.2 m, *P* = 0.07), but there was no difference in predicted VO_2_ max (mean ± SD 39.7 ± 16.2 m vs 38.8 ± 16.0 m, *P* = 0.38). Self-perceived data indicated that 75% of participants preferred the INS intervention and would opt to use this strategy for future exercise.

## Discussion

Only limited studies have evaluated the effects of both reducing insulin and eating carbohydrates as a part of a previous meal without the usual insulin bolus on glycaemic control and performance with aerobic exercise [7, 9–11]. In this study, we compare reducing insulin by 50% as per consensus guidelines and consuming a weight- and exercise intensity-based amount of carbohydrates as per exercise carbohydrates (Excarbs) guidelines [6]. We demonstrate that reducing insulin prior to exercising is likely the preferred strategy for glycaemic control and optimising performance around aerobic exercise in patients with T1D. Furthermore, reducing insulin did not increase the risk of ketoacidosis or lactatemia and there were no episodes of severe hypoglycaemia. Others have also shown that reducing insulin is the best strategy for preventing hyperglycaemia [8, 20], but that consuming extra carbohydrates is the safest option in preventing hypoglycaemia [10, 21]. However, as in this study, athletes with diabetes often prefer the risk of mild hypoglycaemia than hyperglycaemia due to the deleterious effects of the latter on exercise performance [22]. Nevertheless, it is clear that patients with T1D need individualised reductions in their insulin around exercise and the priority in the majority of patients will be preventing hypoglycaemia rather than optimising performance [7]. In particular, patients that are keen to administer insulin around exercise need to ensure adequate monitoring of their glucose levels and have appropriate treatment for hypoglycaemia readily accessible [7].

Despite only being a small pilot study, strengths of this study include that participants acted as their own controls in a direct comparison of the two main approaches in T1D and that unlike other studies, both groups had similar glucose levels at baseline. However, this study did not include women (to minimise the effects of the sex hormones), children or the elderly, and further larger studies are required in both male and female patients across the life span. Future larger studies are also required to determine whether reducing insulin is associated with a significant improvement in aerobic performance when compared with eating additional carbohydrates. Additionally, it will be important to determine whether eating lesser amounts of carbohydrates than in this study can prevent exercise-induced hypoglycaemia and significant hyperglycaemia. Indeed, consensus guidelines recommend that only 10 g of carbohydrate may be sufficient to prevent hypoglycaemia in most patients with T1D who are exercising for less than 1 h [6].

Other limitations of the current study and areas for future research include the implementation of a control trial using a typical day without additional carbohydrate consumption or insulin reduction, and also the use of a validated questionnaire to evaluate perceived performance and preference. All participants in this study also had good glycaemic control, so it would be worthwhile comparing these guideline recommendations in patients with suboptimal glycaemic control, because in many of these patients reductions in insulin or extra carbohydrates may not be required, particularly with short periods of exercise. Lastly, the recommendations will likely differ depending on the different types, intensity, and length of exercise.

## Data Availability

The datasets used and/or analysed during the current study are available from the corresponding author on reasonable request.

## Abbreviations

T1D: Type 1 diabetes
6MWT: 6-minute walk test
INS: Reduced insulin intervention
CARBS: Additional carbohydrates intervention
CSII: Continuous subcutaneous infusion of insulin
VO_2_ max: Maximal oxygen consumption
RPE: Rate of perceived exertion
WRDS: Waikato Regional Diabetes Service
HbA1c: Glycated haemoglobin
FGM: Flash glucose monitor/monitoring
